# Natural Transmission of *Plasmodium berghei* Exacerbates Chronic Tuberculosis in an Experimental Co-Infection Model

**DOI:** 10.1371/journal.pone.0048110

**Published:** 2012-10-26

**Authors:** Ann-Kristin Mueller, Jochen Behrends, Kristine Hagens, Jacqueline Mahlo, Ulrich E. Schaible, Bianca E. Schneider

**Affiliations:** 1 Department of Molecular Infection Biology, Research Center Borstel, Borstel, Germany; 2 Department of Infectious Diseases, Parasitology Unit, University Hospital Heidelberg, Heidelberg, Germany; University of São Paulo, Brazil

## Abstract

Human populations are rarely exposed to one pathogen alone. Particularly in high incidence regions such as sub-Saharan Africa, concurrent infections with more than one pathogen represent a widely underappreciated public health problem. Two of the world’s most notorious killers, malaria and tuberculosis, are co-endemic in impoverished populations in the tropics. However, interactions between both infections in a co-infected individual have not been studied in detail. Both pathogens have a major impact on the lung as the prime target organ for aerogenic *Mycobacterium tuberculosis* and the site for one of the main complications in severe malaria, malaria-associated acute respiratory distress syndrome (MA-ARDS). In order to study the ramifications caused by both infections within the same host we established an experimental mouse model of co-infection between *Mycobacterium tuberculosis* and *Plasmodium berghei* NK65, a recently described model for MA-ARDS. Our study provides evidence that malaria-induced immune responses impair host resistance to *Mycobacterium tuberculosis*. Using the natural routes of infection, we observed that co-infection exacerbated chronic tuberculosis while rendering mice less refractory to *Plasmodium.* Co-infected animals presented with enhanced inflammatory immune responses as reflected by exacerbated leukocyte infiltrates, tissue pathology and hypercytokinemia accompanied by altered T-cell responses. Our results - demonstrating striking changes in the immune regulation by co-infection with *Plasmodium* and *Mycobacterium* - are highly relevant for the medical management of both infections in humans.

## Introduction

Today, 3.3 billion people live in malaria endemic areas with sub-Saharan Africa being one of the most affected regions in the world (WHO). *Plasmodium falciparum* causes the most serious form of the disease with the highest rates of complications and mortality. In sub-Saharan Africa, malaria is co-endemic with tuberculosis in most regions. Despite this obvious epidemiological overlap, very little is known about interactions between the malaria parasite and the tubercle bacillus.

Tuberculosis is the most prevalent bacterial infectious disease in humans. The causative agent, *Mycobacterium tuberculosis,* is transmitted from infected people by aerosols and establishes infection in the lung, from where it can spread to any organ through blood or the lymphatic system. After entering the lung, *M. tuberculosis* is phagocytosed by alveolar macrophages, which provide a niche for its survival and replication and initiate a local inflammatory reaction, while the pathogen is transported to the draining lymph node to induce an antigen-specific T cell response [1], [2]. IFN-γ, the hallmark Th1 cytokine, is central in protection against tuberculosis by activation of macrophages to generate microbicidal effectors and pro-inflammatory cytokines, such as tumor necrosis factor alpha (TNF-α) which contributes to macrophage activation, granuloma formation and control of mycobacterial infection [3], [4]. This initial immune response leads to protective immunity in more than 90% of the infected people, but usually fails to achieve sterile eradication of the pathogen leading to latent infection without clinical signs of tuberculosis. Reactivation can occur after years or decades leading to active tuberculosis. The risk of reactivation increases with conditions that modulate the immune status of the host such as disease, drug treatment, age, malnutrition or stress.

Together with HIV and tuberculosis, malaria comprises the triad of main infectious threats to humankind. Malaria is a vector-borne disease caused by the protozoan parasite *Plasmodium* and is naturally transmitted by the bite of a female *Anopheles* mosquito. Each year, approximately 350–500 million cases of malaria lead to the death of 1–3 million people, predominately young children and pregnant women in sub-Saharan Africa [5].

Severe malaria is often complicated by malaria-associated acute respiratory distress syndrome (MA-ARDS), characterized by pulmonary inflammation, edema and hemorrhages [6]. MA-ARDS is more common in adults than in children, with a higher prevalence in pregnant women and non-immune individuals [7]. As tuberculosis is primarily a disease of the lung, MA-ARDS may affect the course of tuberculosis in co-infected patients. However, no data on the clinical outcome of tuberculosis in people with MA-ARDS are available to date. The rodent malaria parasite *Plasmodium berghei* NK65 (*Pb*NK65) has recently been identified as an experimental model for MA-ARDS [7]. Co-infection with *M. tuberculosis* and *Pb*NK65 therefore is a highly relevant model to study the effects of malaria-induced pulmonary pathology on the outcome of tuberculosis.

Of note, experimental *Plasmodium-Mycobacterium* co-infection studies have been exclusively carried out by infecting mice with parasitized erythrocytes, giving rise to blood-stage malaria infection while excluding the clinically-silent liver-stage phase. However, the liver stage is an obligatory step during infection and relevant for anti-plasmodial immunity [8]–[11]. Here we report on a novel experimental model system to study malaria-tuberculosis co-infection in mice after challenge with both pathogens via their natural routes. Using this model, we observed exacerbated lung pathology, hypercytokinemia and dysregulated T cell responses together with significantly increased mycobacterial loads in co-infected animals, demonstrating that immunity to *M. tuberculosis* was severely compromised after naturally transmitted malaria infection.

## Results

### Co-infected Mice are More Refractory to *Plasmodium* Sporozoite Infection but Exacerbate Tuberculosis

To investigate whether co-infection with *Pb*NK65 affects the outcome of *M. tuberculosis* infection, C57BL/6 mice were infected via the aerosol route with 100 CFU *M. tuberculosis* H37Rv per lung, and 40 days later, when *M. tuberculosis* infection had reached the chronic phase, mice were challenged with *Pb*NK65 sporozoites by mosquito bite. Approximately 10 sporozoite-infected mosquitoes were feeding on each mouse, which resulted in a 100% infection rate as confirmed by the presence of blood-stage parasites 4–5 days later (Table 1).

**Table 1:** Prepatency after sporozoite transmission by mosquito bite.

Experiment	Experimental mouse group (C57BL/6)	Challenge by 10 infectious mosquito bites	No. of blood-stage positive animals/No. of animals pre group	Mean prepatency[d]
**#1**	naïve	*Pb*NK65	7/7	4,4
	Mtb infected	*Pb*NK65	7/7	5,5
**#2**	naïve	*Pb*NK65	20/20	5,5
	Mtb infected	*Pb*NK65	20/20	5,5

All mice developed blood-stage infections after natural transmission of PbNK65 sporozoites by mosquito bite. Data from two independent experiments are shown.

**Figure 1:**
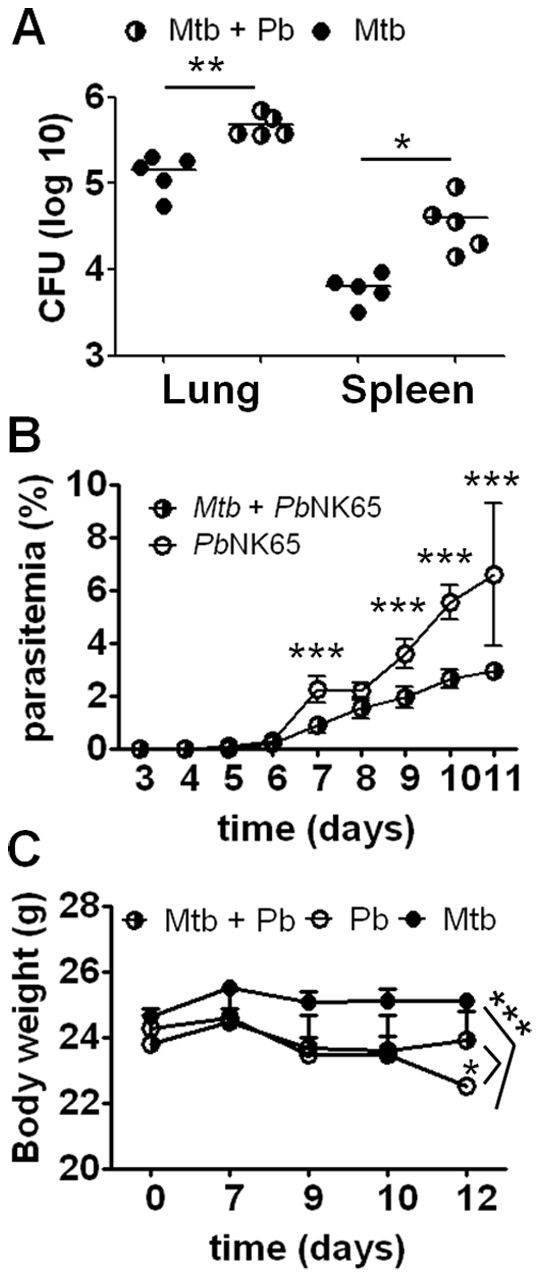
Co-infected mice are more refractory to *Plasmodium* sporozoite infection but exacerbate tuberculosis. C57BL/6 mice were aerosol infected with *M. tuberculosis* H37Rv (100 CFU/lung) and 40 days later challenged with *Pb*NK65 sporozoites by mosquito bite. Control mice were infected with *M. tuberculosis* or *Pb*NK65 alone, respectively. A) Mice were sacrificed 12 days after co-infection and serial dilutions of lung and spleen lysates were plated for CFU determination. B) Parasitemia in peripheral blood was monitored by daily Giemsa-stained blood smears. Note, that co-infected animals had significantly lower parasite numbers than mice infected with *Pb*NK65 alone. Results are shown as means ± SD (n = 10). C) Weight loss was reduced when mice were pre-infected with *M. tuberculosis* before *Pb*NK65 challenge. Results are shown as means ± SD (n = 10). Results from one representative experiment out of two independent ones are shown. Statistical analysis was performed using the Student’s *t* test (A) or ANOVA (B and C) (*p<0.05; **p<0.01; *** p<0.001).

Twelve days post co-infection the mycobacterial loads in lungs and spleens were significantly increased in animals co-infected with *Pb*NK65 (Fig. 1 A), indicating that malaria caused reactivation of chronic tuberculosis. While co-infection promoted *M. tuberculosis* infection, it led at the same time to better control of *Pb*NK65 sporozoite infection as reflected by significantly lower parasite levels in peripheral blood (Fig. 1 B) and less body weight loss (Fig. 1 C) as compared to animals infected with *Pb*NK65 alone.

### Severe Inflammation in Lungs of Mice Co-infected with *M. tuberculosis* and *Pb*NK65

**Figure 2:**
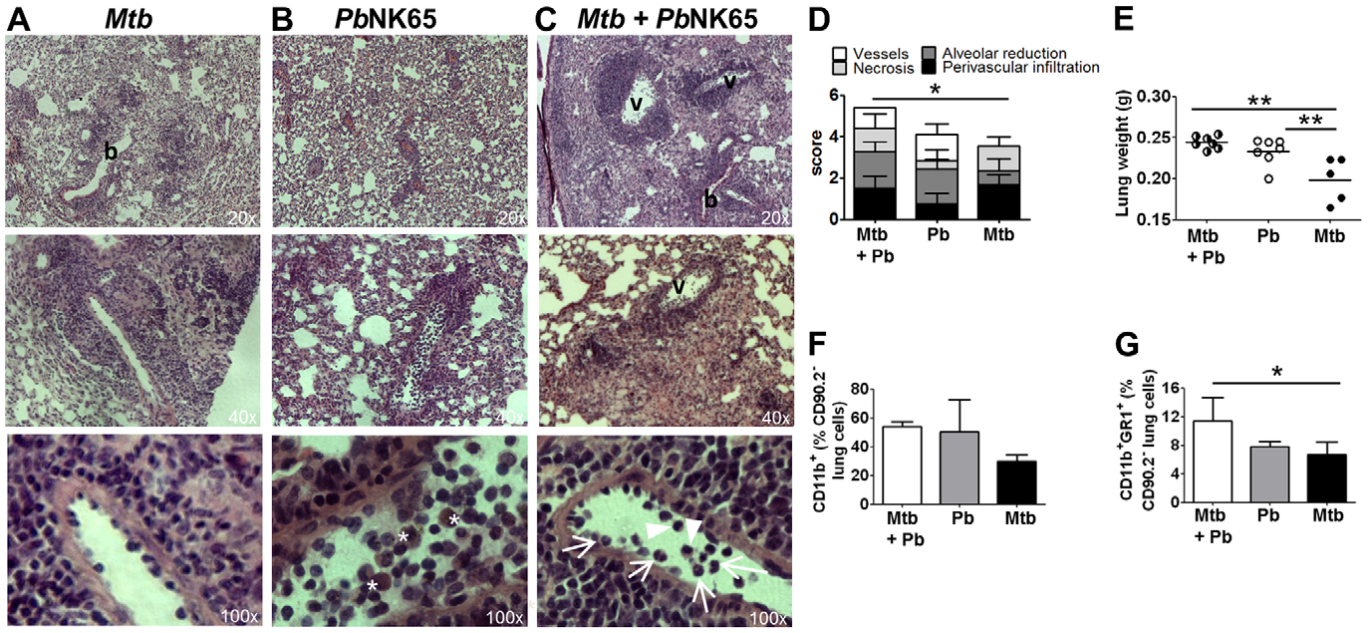
Malaria co-infection increases inflammatory tissue responses in *M. tuberculosis* infected lungs. C57BL/6 mice were aerosol infected with *M. tuberculosis* H37Rv (100 CFU/lung) and 40 days later challenged with *Pb*NK65 sporozoites by mosquito bite. Control mice were infected with *M. tuberculosis* or *Pb*NK65 alone, respectively. A–C) Representative H&E stains of lungs 13 days after co-infection. Note, that *Pb*NK65 co-infection exacerbated tissue pathology compared to lungs of mice infected with *M. tuberculosis* alone (v  =  vessel, b  =  bronchus; asterisks indicate hemozoin loaded cells; arrow: neutrophils; arrowhead: monocytes). D) Histopathological scores from co-infected, *Pb*NK65 or *M. tuberculosis* infected lungs are shown. Pathology was most severe in co-infected animals, with the total score being significantly increased compared to *M. tuberculosis* infected lungs (n = 4 for co-infected and *Pb*NK65 infected; n = 5 for *M. tuberculosis* infected). E) Lung weights 12 days after co-infection. F–G) Lung leukocytes were analyzed for surface expression of CD11b and GR-1. Results are shown as means ± SD (n = 3–5). Results from one representative experiment out of two independent ones are shown. Statistical analysis was performed by ANOVA (*p<0.05; **p<0.01).

Histopathology of the lungs revealed cellular infiltration as well as hemorrhage (Fig. 2 B) in *Pb*NK65 infected animals as described before [12]. Hence, when *M. tuberculosis* infected mice were co-infected with *Pb*NK65 they presented with more extensive pulmonary leukocyte infiltrations and increased total lung weight than after *M. tuberculosis* infection alone (Fig. 2 A–E). High numbers of leukocytes were observed marginating along vessel walls and infiltrating the *M. tuberculosis* infected lung tissue upon *Pb*NK65 co-infection, with polymorphonuclear neutrophils and monocytes being the most abundant cell types (Fig. 2 C lower panel, arrows and arrowheads). Alveolar reduction was much more pronounced in the presence of *Pb*NK65 while tissue necrosis was comparable in co-infected and *M. tuberculosis* infected lungs (Fig. 2 D). Frequently, macrophages with abundant malaria pigment (hemozoin) were observed in *Pb*NK65 infected lungs (Fig. 2 B lower panel, asterisks), which were however less frequent in co-infected animals reflecting the lower parasitemia levels in those animals.

FACS analysis of lung leukocytes revealed an increase in CD11b^+^ cells in co-infected compared to *M. tuberculosis* infected mice (Fig. 2 F and Figure S1 A). Of those, GR1^+^ neutrophils were significantly increased in co-infected compared to *M. tuberculosis* infected lungs (Fig. 2 G and Figure S1 A), confirming the histology.

**Figure 3:**
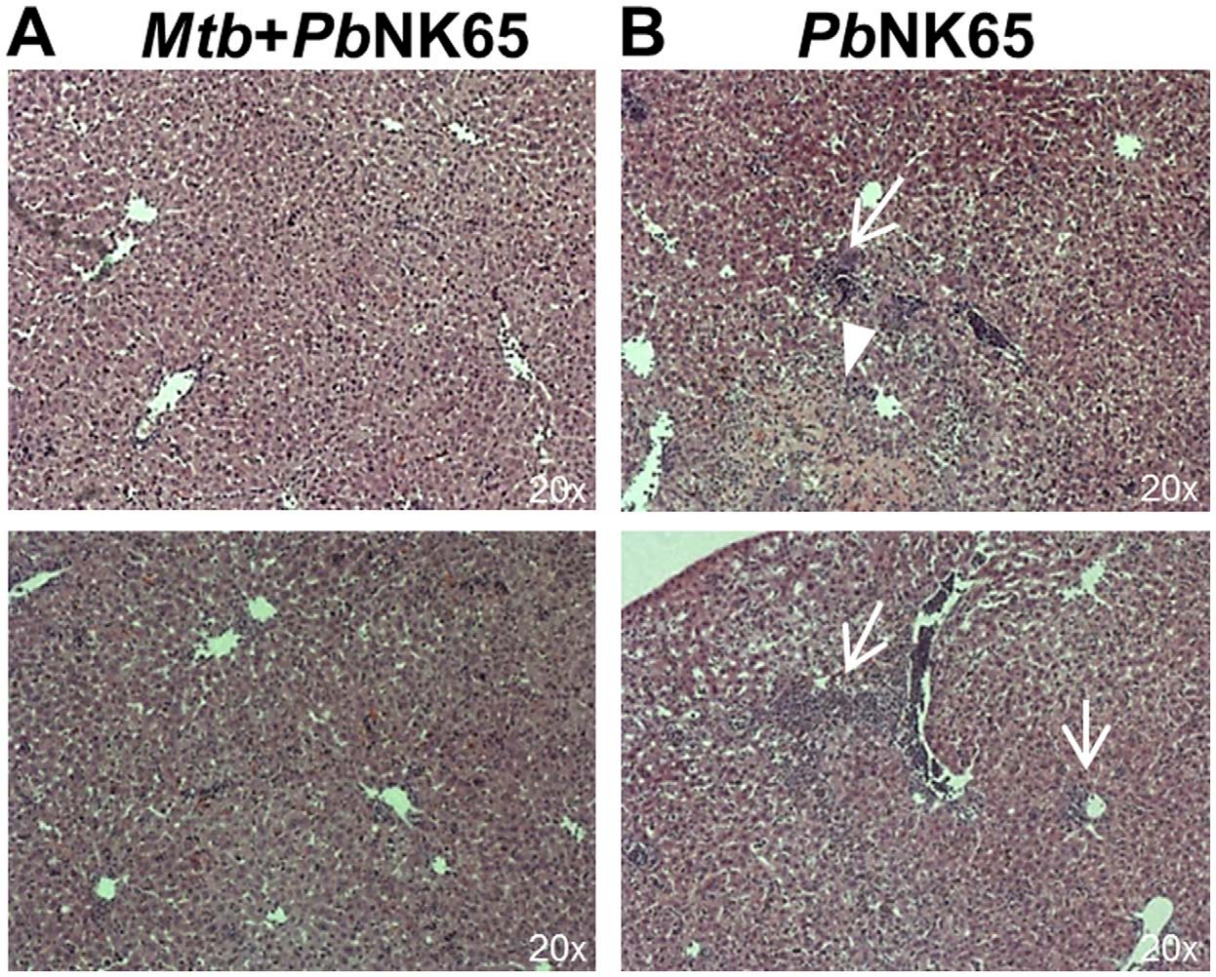
*Pb*NK65 associated liver damage is reduced in co-infected mice. C57BL/6 mice were aerosol infected with *M. tuberculosis* H37Rv (100 CFU/lung) and 40 days later challenged with *Pb*NK65 sporozoites by mosquito bite. Control mice were infected with *M. tuberculosis* or *Pb*NK65 alone, respectively. Representative H&E stains of liver sections 13 days after co-infection. Note, that *Pb*NK65 infection caused periportal inflammation (B; arrows) and tissue necrosis (B; arrowhead) which was reduced in livers of co-infected animals (A).

Histological analysis of the liver revealed some periportal inflammation and tissue necrosis in *Pb*NK65 infected animals which however, was reduced in animals co-infected with *M. tuberculosis* (Fig. 3 A and B). Reduced liver pathology together with a reduction in peripheral blood parasitemia (Fig. 1 B) indicates an increased resistance to *Pb*NK65 infection in *Mtb* infected animals.

Taken together, *Pb*NK65 induced leukocyte infiltration led to exacerbated inflammation and tissue pathology in *M. tuberculosis* infected lungs.

### Impact of *Plasmodium-Mycobacterium* Co-infection on T Cell Responses in Lung, Spleen and Liver

**Figure 4:**
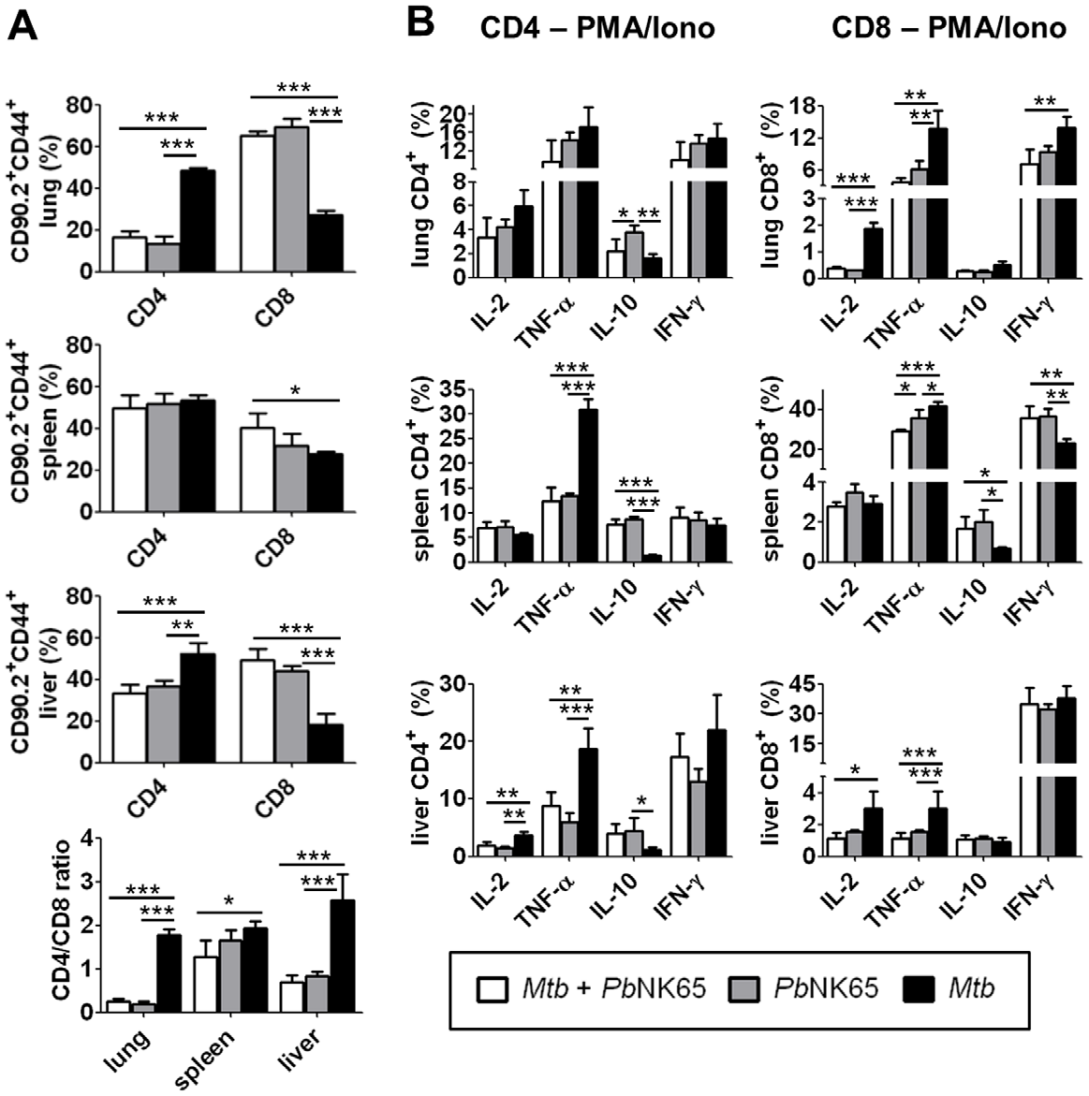
*Pb*NK65 co-infection alters T cell responses in *M. tuberculosis* infected mice. C57BL/6 mice were infected by aerosol with *M. tuberculosis* H37Rv (100 CFU/lung) and challenged with *Pb*NK65 sporozoites by mosquito bite 40 days later. Control mice were infected with *M. tuberculosis* or *Pb*NK65 alone, respectively. A) 12 days upon *Pb*NK65 infection, lungs, spleens and livers were analyzed for the presence of CD44 positive CD4 and CD8 effector T cells by flow cytometry. B) Whole lung and spleen lysates and purified liver lymphocytes were re-stimulated *in vitro* with PMA/Iono (50 ng/ml, respectively) and analyzed by flow cytometry for the presence of IL-2, TNF-α, IL-10 or IFN-γ producing CD4 and CD8 T cells. Results are shown as means ± SD (n = 3–5). Data from one out of two independent experiments are shown. Statistical analysis was performed by ANOVA (*p<0.05; **p<0.01; *** p<0.001).

The histopathological alterations observed in co-infected mice indicate that *Pb*NK65 modulates inflammatory immune responses to *M. tuberculosis*. To assess whether T cell responses were affected by malaria co-infection, lung, spleen and liver cells from co- and single-infected animals were analyzed by FACS. Frequencies of CD4 and CD8 T cells were significantly altered upon co-infection in all three organs when compared to mice infected with *M. tuberculosis* alone (Fig. 4 A). While the frequencies of CD4 T cells were, except from the spleen, significantly decreased upon co-infection with *Pb*NK65, frequencies of CD8 T cells were significantly increased, resulting in a reversed CD4/CD8 T cell ratio.

Functional analysis of CD4 and CD8 T cells recruited to the respective organs by intracellular cytokine staining upon *ex vivo* re-stimulation revealed a shift in the balance of T cell derived TNF-α/IL-10 towards IL-10 in co-infected animals (Fig. 4 B and Figure S1 B). Particularly in spleen and liver, the frequencies of TNF-α producing CD4 and CD8 T cells were dramatically decreased while the percentage of IL-10 producers was significantly elevated compared to those of *M. tuberculosis* infected animals (Fig. 4 B middle and lower panel). In the lung, CD8 but not CD4 T cells were subject to *Plasmodium*- induced immunomodulation, as similar percentages of pulmonary CD4 T cells but significantly reduced frequencies of CD8 T cells produced IL-2, TNF-α or IFN-γ in co-infected compared to *M. tuberculosis* infected mice (Fig. 4 B upper panel). In addition, IFN-γ producing CD8 T cell frequencies were increased in spleens from co-infected mice (Fig. 4 B middle panel) and frequencies of hepatic IL-2 producing CD4 and CD8 T cells were reduced upon co-infection in comparison to those infected with *M. tuberculosis* only (Fig. 4 B lower panel). Of note, overall cytokine profiles of CD4 and CD8 T cells from co-infected animals were comparable to those from animals solely infected with *Pb*NK65, indicating that *Pb*NK65 overwrites T-cell responses induced in mice only infected with *M. tuberculosis*.

### Cytokine Overproduction (Hypercytokinemia) in Mice Co-infected with *M. tuberculosis* and *Pb*NK65

**Figure 5:**
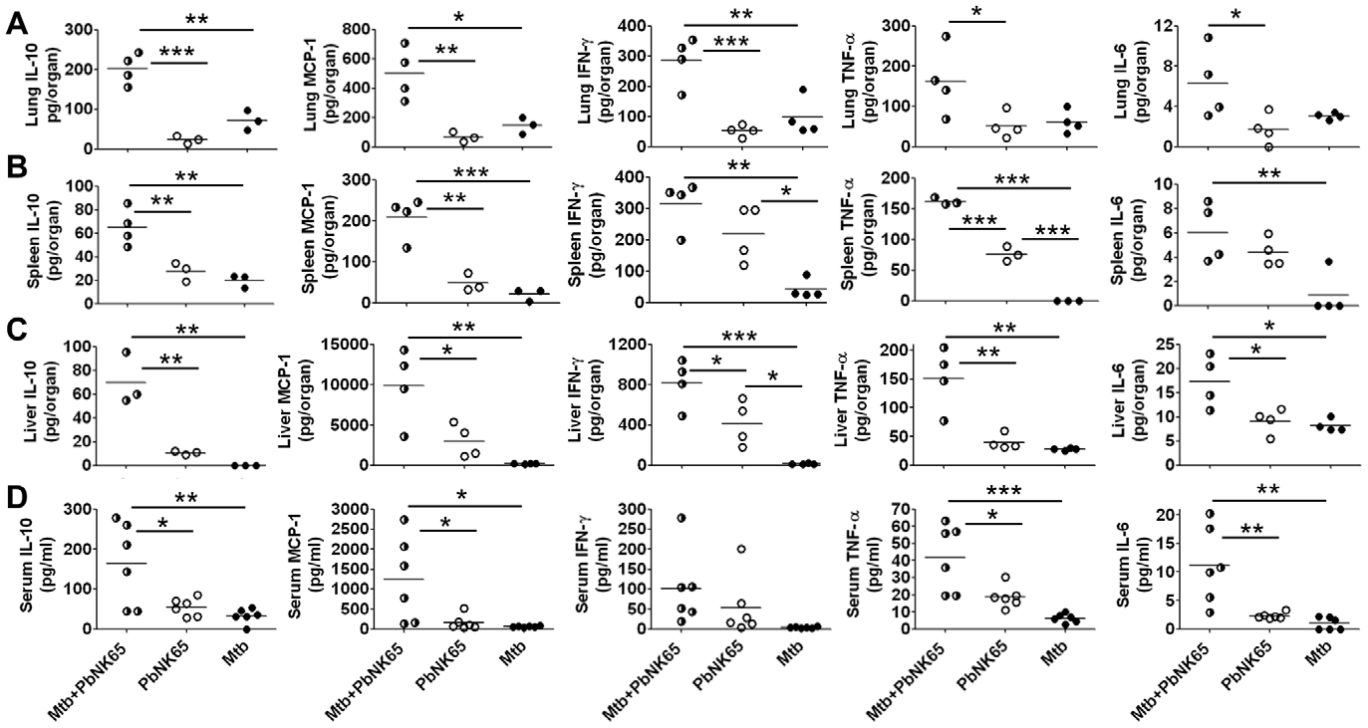
Co-infection with *M. tuberculosis* and *Pb*NK65 induces a cytokine storm. C57BL/6 mice were infected by aerosol with *M. tuberculosis* H37Rv (100 CFU/lung) and challenged with *Pb*NK65 sporozoites by mosquito bite 40 days later. Control mice were infected with *M. tuberculosis* or *Pb*NK65 alone, respectively. Cytokine levels were measured in lungs (A), spleens (B), livers (C) and sera (D) 13–14 days after co-infection. Statistical analysis was performed by ANOVA and Tukey’s Multiple Comparison test. (*p<0.05; **p<0.01; ***p<0.001). Data from one out of two independent experiments are shown.

Alterations in leukocyte recruitment and T cell cytokine production in malaria-tuberculosis co-infected mice prompted us to investigate cytokine protein levels in tissue and serum of those animals. We found significantly elevated protein concentrations of IL-10, monocyte chemoattractant protein-1 (MCP-1), IFN-γ, TNF-α and IL-6 in lungs, spleens, livers as well as sera of malaria-tuberculosis co-infected mice (Fig. 5 A–D). Highly elevated levels of cytokines primarily produced by macrophages and dendritic cells (IL-6, MCP-1, TNF-α, IL-10) in *M. tuberculosis*-*Pb*NK65 co-infected animals indicate that immune regulation is significantly impaired when malaria is concurrent with tuberculosis in mice.

## Discussion

Using *Pb*NK65, a rodent *Plasmodium* strain that has been described as a new model for MA-ARDS in C57BL/6 mice [12], we have established a novel experimental model system to study the effect of malaria associated lung pathology on tuberculosis in the mouse. The advantage of *Pb*NK65 over the infection of mice with *Pb*ANKA, another rodent malaria parasite known to cause pulmonary complications such as acute lung injury and ARDS [13], is the fact that *Pb*NK65 infected C57BL/6 mice survive significantly longer compared to *Pb*ANKA infected mice. *Pb*ANKA is the prime model for cerebral malaria [14]. Susceptible C57BL/6 mice infected with *Pb*ANKA die very rapidly from experimental cerebral malaria usually within 6–8 days ([15] and our own observations). This time window is too narrow to detect any potential effects of malaria infection on the containment or replication of *M. tuberculosis*. Therefore, we found the *Pb*NK65 infection model more appropriate to study the effect of malaria induced lung pathology on the outcome of *M. tuberculosis* infection. Importantly, in order to experimentally reproduce aspects of both diseases more precisely as it occurs during naturally acquired human infections, mice were challenged with both pathogens via their natural routes of infection, i.e. aerosol and mosquito bite, respectively.

In our model, *Pb*NK65 co-infection of mice chronically infected with *M. tuberculosis* exacerbated tuberculosis. Upon co-infection with naturally transmitted *Pb*NK65, mice failed to control *M. tuberculosis* replication and presented with more severe lung pathology as characterized by massive influx of inflammatory leukocytes including neutrophils. Although neutrophils are the most commonly infected phagocytes in patients with active tuberculosis [16], their role is controversially discussed. There is however, good evidence that neutrophils play only a minor role restricting or eliminating *M. tuberculosis* but rather contribute to pathology and tissue destruction [17]–[20]. While they seem to contribute to very early defense against mycobacteria [21]–[25], massive presence of neutrophils in *M. tuberculosis* infection have been associated with poor disease outcome in both, humans and mice [26]–[30]. Increased neutrophil influx together with increased total leukocyte recruitment therefore indicates loss of control of inflammation in co-infected mice.

Highly elevated cytokine levels in tissues and sera of co-infected mice further indicate impaired immune regulation. Both, *M. tuberculosis* and *Pb*NK65 induce Th1 immune responses, which in concert may cause an overwhelming pro-inflammatory cytokine storm difficult to be counter-regulated similar to what has been observed in sepsis [31]–[35]. IL-10 levels are also significantly enhanced in co-infected mice like in sepsis where it is associated with a compensatory anti-inflammatory response. IL-10 is a negative regulator of Th1 responses and of central importance in immunity to malaria, where it ameliorates immunopathology at the expense of parasite elimination [36]–[39]. However, high levels of IL-10 can result in profound immunosuppression. It has been shown that IL-10 deactivates macrophage effector function against intracellular pathogens [40]–[43]. In fact, IL-10 antagonizes pro-inflammatory responses essential for protective immunity to *M. tuberculosis* [44]–[46] such as phagosome maturation [47] and IFN-γ induced production of reactive nitrogen intermediates [42], which mediate the killing of *M. tuberculosis* [48], [49]. Importantly, both macrophage and T cell derived IL-10 suppress macrophage function in mycobacterial infection [50], [51]. IL-10 not only counteracts IFN-γ mediated macrophage activation but also inhibits macrophage programmed cell death [52]–[54], which is an important mechanism to eliminate intracellular mycobacteria [48], [55], [56]. Macrophage deactivation by IL-10 has been shown to exacerbate disease [50] and to be associated with reactivation of tuberculosis in humans and mice [57]–[59]. In line with this, despite high levels of both IFN-γ and TNF-α, co-infected mice were unable to control *M. tuberculosis* replication. Overproduction of IL-10 associated with concurrent *Pb*NK65 infection is therefore likely to exacerbate the outcome of tuberculosis by interfering with macrophage associated bacterial clearance.

Apart from its role in macrophage activation, TNF-α is a central mediator of granuloma formation and plays a major role in the control of persistent *M. tuberculosis* infection [3]. However, tight regulation of TNF-α production is required to protect the host from its detrimental activities. Both, loss and overproduction of TNF-α have fatal effects on the outcome of tuberculosis, be it due to a loss of granuloma structure and excessive pathology or due to impaired macrophage activation [60], [61]. TNF-α directly effects immune cell recruitment by upregulation of endothelial adhesion molecules [62] and induction of chemokine production which further recruit leukocytes to the site of infection [63], [64]. Hence, overproduction of TNF-α in co-infected mice most likely contributes to exacerbated inflammation and immunopathology.

CD4 and CD8 T cell responses are significantly altered in co-infected mice when compared to those infected with *M. tuberculosis* alone. Of note, the profile of the T cell response in co-infected animals resembles that of animals infected with *Pb*NK65 alone, indicating that *Pb*NK65 infection overwrites the *M. tuberculosis* biased T cell responses. CD8 T cells play a major role in cellular immunity to malaria as reflected by increased frequencies of CD8 T cells in co-infected compared to *M. tuberculosis* infected animals. Moreover, particularly in spleen and liver, the frequencies of TNF-α producing CD4 and CD8 T cells were dramatically decreased while the percentage of IL-10 producers was significantly elevated, shifting the balance of T cell derived TNF-α/IL-10 towards IL-10. Hence, *Plasmodium* co-infection modulated established immune responses triggered by *M. tuberculosis* infection with a detrimental impact on the outcome of tuberculosis.

Higher susceptibility to tuberculosis upon concurrent *Plasmodium* infection has been described before [65], [66]. Those studies were carried out exclusively using parasitized erythrocytes for infection, and only one study used virulent *M. tuberculosis,* while the other one studied co-infection between *Plasmodium* and BCG, the tuberculosis vaccine strain. More importantly, neither *P. yoelii* nor *P. chabaudi* used in those studies cause MA-ARDS in mice as observed upon *Pb*NK65 infection. This explains the more pronounced differences in pathology and bacterial burden observed herein when compared to other studies. We believe that our co-infection model is more relevant to the human situation as it not only mimics the natural course of malaria (by mosquito bite transmission) and tuberculosis infections, but also takes MA-ARDS into account. Of note, our study is the only one considering the fact that natural infection by mosquito bite is more efficient than simulated infection via needle infections (e.g. iv injections of malarial sporozoites) [67].

Malaria-tuberculosis co-infected mice were more resistant to *Pb*NK65 infection as reflected by reduced parasitemia levels, liver pathology and body weight loss. Non-specific protection to *Plasmodium* infection by mycobacteria has been reported before [68]–[70]. Presumably, mycobacteria-elicited pro-inflammatory responses protect mice against subsequent *Plasmodium* infection. One possible mechanism could be IFN-γ and TNF-α mediated activation of macrophages, which contribute to parasite clearance particularly in blood and spleen of infected animals. Chronically *M. tuberculosis* infected mice may already carry pre-activated macrophages at the time of *Plasmodium* infection, which most likely accelerate clearance of malaria parasites. This is in line with the observation that the development of blood-stage parasitemia following *Pb*NK65 sporozoite transmission was delayed in animals pre-infected with *M. tuberculosis* and, upon appearance of parasites in the periphery, blood parasite levels were significantly reduced compared to those in mice infected with *Pb*NK65 alone.

Together our data suggest an overly aggressive innate response in co-infected animals, with severely increased and uncontrolled recruitment of pro-inflammatory leukocytes to the lung and hypercytokinemia. The fact that Th1 T cell responses are rather decreased in co-infected animals, with an enhanced ratio of IL-10 to IFN-γ/TNF-α producing T cells, suggests that the cytokine storm is rather caused by uncontrolled cytokine secretion from innate immune cells such as macrophages.

In summary, our data demonstrate that immune responses to tuberculosis and malaria mutually influence each other during co-infection. A recent clinical study in Guinea-Bissau seems to support our data as it reported improved clinical outcome and reduced mortality among severely ill tuberculosis patients after malaria prevention had been carried out [71]. In conclusion, our data indicate that timely diagnosis of malaria-tuberculosis co-infection and MA-ARDS is critical for the clinical management of co-infected individuals and the control of exacerbated lung pathology in tuberculosis patients.

## Materials and Methods

### Ethics Statement

Animal experiments were approved by the Ethics Committee for Animal Experiments of the Ministry for Agriculture, Environment, and Rural Areas of the State of Schleswig-Holstein (Kommission für Tierversuche/Ethik-Kommission des Landes Schleswig-Holstein) under the license 33–3/10 (“Die Auswirkung von Tuberkulose auf die Pathogenese und Immunantwort bei Malaria im Rahmen einer Koinfektion in der Maus”/”The impact of tuberculosis on pathogenesis and immune responses to malaria in an experimental co-infection mouse model”).

### Plasmodium Life Cycle

*Anopheles stephensi* mosquitoes were raised at 28°C, 75% humidity under a 12-h light/12-h dark cycle and maintained on a 10% sucrose/PABA solution during adult stages. 4–5-day-old female mosquitoes were blood-fed on anaesthetized NMRI mice that had been infected with *Plasmodium berghei* NK65. Rodents were assayed for high levels of parasitemia and the abundance of gametocyte-stage parasites capable of exflagellation. After the infective blood meal, mosquitoes were maintained at 21°C, 80% humidity. On day 10 post feeding, mosquitoes were dissected in RPMI 1640 medium, and isolated midguts were examined for the infection rate. Infectious salivary gland sporozoite populations were separated as described previously [72], [73].

### Bacterial Strains and Culture

*M. tuberculosis* H37Rv was grown in Middlebrook 7H9 broth (BD Biosciences) supplemented with 0.5% glycerol and asparagine (1 g/l), and OADC (Oleic acid, Albumin, Dextrose, Catalase) enrichment medium (BD). Bacterial cultures were harvested and aliquots were frozen at –80°C until later use. Viable cell counts in thawed aliquots were determined by plating serial dilutions of cultures onto Middlebrook 7H11 agar plates followed by incubation at 37°C.

### Mice, Infection and Colony Forming Units (CFUs)

For all experiments female C57BL/6 mice aged between 6–8 weeks were used, which were obtained from Charles River Laboratories. Mice were maintained under specific barrier conditions in BSL 3 facilities.

For infection of experimental animals, *Mycobacterium tuberculosis* stocks were diluted in sterile distilled water/1% v/v Tween-80/1% w/v albumin at a concentration providing an uptake of 100 viable bacilli per lung. Infection was performed via the respiratory route by using an aerosol chamber (Glas-Col, Terre-Haute, IN, USA). Animals were exposed for 60 min to an aerosol generated by nebulising the prepared *M. tuberculosis* suspension. The inoculum size was quantified 24 h after infection by determining bacterial loads in the lungs of infected mice.

Bacterial loads in lung, spleen and liver were evaluated at different time points after aerosol infection by mechanical disruption of the organs in 0,05% v/v Tween 20 in PBS containing a proteinase inhibitor cocktail (Roche Diagnostics) prepared according to the manufacturer’s instructions. Tenfold serial dilutions of organ homogenates in sterile water/1% v/v Tween 80/1% w/v albumin were plated onto Middlebrook 7H11 agar plates and incubated at 37°C. Colonies were enumerated after 3–4 weeks.

### Natural Malaria Parasite Infection Experiments

Naïve mice or animals pre-infected for 40 days with *M tuberculosis* were infected with *Plasmodium berghei* NK65 by mosquito bite. Mice were exposed for 15 min to highly infected mosquitoes that contained an average of 30,000 wild-type salivary gland sporozoites each. Successful blood feeding was confirmed by mosquito dissection after the co-infection experiment. Parasitemia (occurrence of intraerythrocytic stages) was monitored by daily Giemsa stained blood smears.

### Cell Isolation and Purification from Lungs, Spleens and Livers

Lungs were removed, sectioned and incubated in PBS containing liberase (1,5 mg/ml; Worthington Biochemical) and DNAse I (250 µg/ml; Worthington Biochemical) at 37°C for 90 min. Digested lung tissue was broken into single cell suspension by subsequent passage through a 100 µm pore size nylon cell strainer. Spleens and livers were also passed through a 100 µm pore size nylon cell strainer to obtain single cell suspensions. Liver infiltrating lymphocytes were isolated using a Percoll Gradient. Remaining erythrocytes were lysed and cells were resuspended in RPMI 1640 medium supplemented with 2 mM glutamine, 1% Hepes (v/v), β-mercaptoethanol (50 µM), and 10% v/v heat-inactivated fetal calf serum (complete RPMI 1640 medium).

### Flow Cytometry

For flow cytometric analysis of surface markers and intracellular cytokines, single-cell suspensions of lungs, spleens and livers were stained with optimal concentrations of the following specific antibodies: CD8a-V450, CD8a-FITC, CD4-V500, Gr-1-APC, IL-10-PE, IFN-γ-APC from BD Biosciences, CD11b-PerCP-Cy5.5, CD90.2-PECy7, CD90.2-eFluor780, CD44 PerCP-Cy5.5, TNF-α-eFluor450 and IL-2-PeCy7 from eBioscience, and CD44-FITC from BioLegend. Samples were analyzed on a FacsCantoII® flow cytometer (BD Biosciences) equipped with a 405 nm, 488 nm and 633 nm laser and the FCSExpress software (DeNovo™ Software).

### Intracellular Cytokine Staining

Single cell suspensions of lung, spleen or liver (1×10^6^) were stimulated for 4.5 h with phorbol 12-myristate 13-acetate (PMA) and ionomycin (Iono) (Sigma; 50 ng/ml, respectively) in the presence of GolgiPlug™ (BD Biosciences, contains brefeldin A). Non-specific antibody binding was blocked by incubation with a cocktail containing anti-FcγRIII/II mAb (clone 2.4G2, BioLegend), mouse, hamster and rat serum. Subsequently, cells were stained with directly labelled anti-CD90.2, anti-CD44, anti-CD4 and anti-CD8a antibodies for 20 min at 4°C. After washing, cells were fixed and permeabilized for 20 min with Cytofix/Cytoperm™ (BD Biosciences). Cells were washed with Perm/Wash buffer™ (BD Biosciences), and stained with directly labelled anti-IFN-γ (XMG1.2), anti-IL-10 (JES5-16E3), anti-IL-2 (JES6-5H4) and anti-TNF-α (MP6-XT22) antibodies for 45 min at 4°C.

### Multiplex Cytokine Assays

The concentrations of IL-6, IL-10, MCP-1, IFN-γ, and TNF-α in lung, spleen and liver homogenates and serum were determined by cytometric bead array (Mouse Inflammation Kit, BD Biosciences) according to the manufacturer’s protocol.

### Histology

Organs from infected mice were fixed with 4% w/v PFA for 24 h and embedded in paraffin. Sections (3 µm) were rehydrated by running through xylenes, alcohols of decreasing concentrations and finally water. Sections were stained with hematoxylin and eosin (H&E) and analyzed with an Axioplan 2 light microscope and Volocity software (Perkin Elmer).

Histological sections of infected lungs were scored in a blind manner using the following pathological scores (scores are given in parentheses after each category):

1. Perivascular infiltration: no infiltrates (0), mild (<25%; 1), moderate (25–50%; 2) or severe (>50%; 3) infiltration.
2. Alveolar reduction: no pathological changes (0), mild (1), moderate (2), or severe (3).
3. Necrosis: no necrosis (0), mild (1) or severe (2).
4. Vessels: free (0); luminal myeloid cells (1), massive blockage (2).

### Statistical Analysis

Statistical analysis was performed using ANOVA compared with Tukey`s or Bonferroni post-testing. The unpaired Student’s *t* test was employed to compare bacterial titres.

## Supporting Information

Figure S1 Flow cytometric analysis. C57BL/6 mice were infected by aerosol with *M. tuberculosis* H37Rv (100 CFU/lung) and challenged with *Pb*NK65 infectious sporozoites by mosquito bite 40 days later. Control mice were infected with *M. tuberculosis* or *Pb*NK65 alone, respectively. A) 12 days upon *Pb*NK65 infection, lung leukocytes were analyzed for surface expression of CD11b and GR-1. Representative cell-frequency dot plots of electronically gated CD90.2 negative lung cells stained with anti-CD11b and anti-GR1 are shown, in which the numbers represent the frequencies as percentages. B) T cells in lung, spleen and liver were analyzed for the production of IL-2, TNF-α, IL-10 and IFN-γ. Representative dot plots of electronically gated CD90.2^+^CD8^+^ (left dot plots) or CD90.2^+^CD4^+^ (right dot plots) spleen cells are shown. Numbers represent the frequency of the cells as percentage.
(TIF)
