# Supplementary figures and images for: Natural Transmission of Plasmodium berghei Exacerbates Chronic Tuberculosis in an Experimental Co-Infection Model
Source: PLoS One. 2012 Oct 26;7(10):e48110. doi: 10.1371/journal.pone.0048110 (PMC3482195; doi:10.1371/journal.pone.0048110)

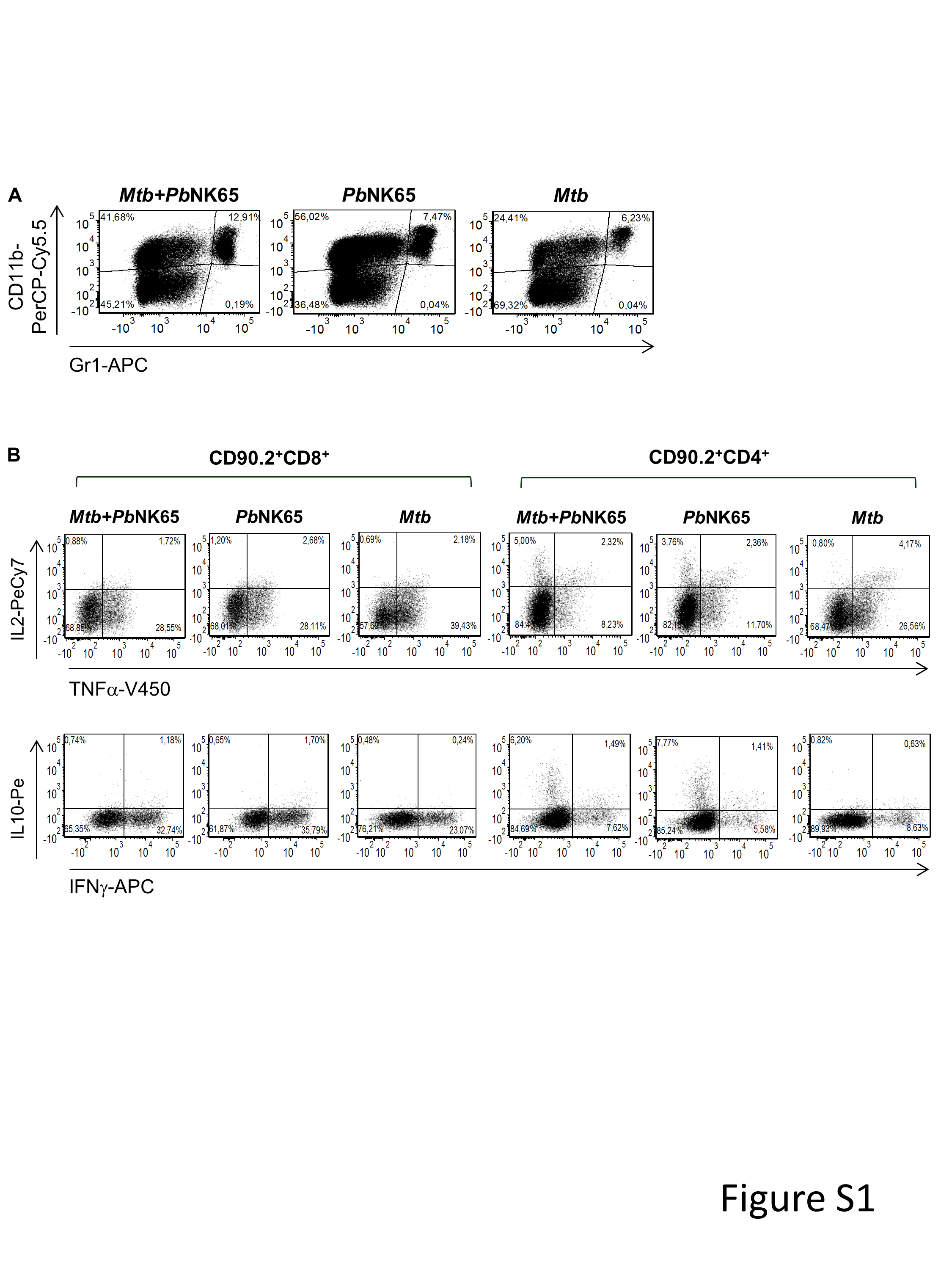

Supplement: Figure S1 — Flow cytometric analysis. C57BL/6 mice were infected by aerosol with M. tuberculosis H37Rv (100 CFU/lung) and challenged with PbNK65 infectious sporozoites by mosquito bite 40 days later. Control mice were infected with M. tuberculosis or PbNK65 alone, respectively. A) 12 days upon PbNK65 infection, lung leukocytes were analyzed for surface expression of CD11b and GR-1. Representative cell-frequency dot plots of electronically gated CD90.2 negative lung cells stained with anti-CD11b and anti-GR1 are shown, in which the numbers represent the frequencies as percentages. B) T cells in lung, spleen and liver were analyzed for the production of IL-2, TNF-α, IL-10 and IFN-γ. Representative dot plots of electronically gated CD90.2+CD8+ (left dot plots) or CD90.2+CD4+ (right dot plots) spleen cells are shown. Numbers represent the frequency of the cells as percentage. (TIF) [file pone.0048110.s001.tif]
